# Infectious Endocarditis Is Associated with Dental Treatment or Poor Dental Status—Results from the Brandenburg Endocarditis Registry (B.E.R.)

**DOI:** 10.3390/jcm14082784

**Published:** 2025-04-17

**Authors:** Roya Ostovar, Anna-Maria Necaev, Filip Schröter, Farzaneh Seifi Zinab, Gesine Dörr, Gerhard Schmalz, Johannes Maximillian Albes

**Affiliations:** 1 Department of Cardiac and Vascular Surgery, Heart Center Brandenburg, University Hospital Brandenburg Medical School, Faculty of Health Sciences Brandenburg, Ladeburger Str. 17, 16321 Bernau bei Berlin, Germany; anna-maria.necaev@immanuelalbertinen.de (A.-M.N.); filip.schroeter@immanuelalbertinen.de (F.S.); johannes.albes@immanuelalbertinen.de (J.M.A.); 2 Department of Cardiac Surgery, Sana Heart Center, 03048 Cottbus, Germany; farinaz.medicine@gmail.com; 3 Department of Cardiology, Alexianer St. Josef’s Hospital, 14471 Potsdam, Germany; g.doerr@alexianer.de; 4 Department of Conservative Dentistry and Periodontology Brandenburg Medical School, Faculty of Sciences Brandenburg, 14776 Brandenburg an der Havel, Germany; gerhard.schmalz@mhb-fontane.de

**Keywords:** dental care, infectious endocarditis, oral hygiene, cardiac surgery, viridans Streptococci, Staphylococcus areus, Enterococcus faecalis

## Abstract

**Background:** While the relationship between recent dental treatment and the development of endocarditis is largely undisputed, the relationship between poor dental status and the development of infective endocarditis has not yet been proven beyond doubt. We have therefore analyzed this hypothetical connection using our established endocarditis register (B.E.R.). **Patients and Methods:** A total of 72 patients who underwent dental treatment (TREAT) and 55 patients with a desolate dental status (DESOLATE) were found in our database of 530 patients subsequently developing infective endocarditis necessitating valve surgery. A propensity score analysis was performed comparing TREAT as well as DESOLATE with matched patients without these conditions as CONTROL. **Results:** TREAT showed significantly more often Strept. mitis (26.9%) as well as other Streptococci (36.54%, *p* = 0.001) compared to CONTROL (3.51% and 10.53%, respectively), whereas Staphylococci and E. faecalis were found more often in CONTROL than in TREAT (*S. aureus*: 22.81% vs. 15.38%, n.s.; other Staphylococci 14.04% vs. 1.92%, *p* = 0.033; E. faec.: 26.32% vs. 9.62%, *p* = 0.045). DESOLATE showed significantly more Strept. mitis compared to CONTROL (27.91% vs. 4.88%, *p* = 0.007). Early mortality was 23.7% in the TREAT group, while it was 15.25% in the CONTROL group and 17.02% in the DESOLATE group vs. 20.83% in the CONTROL patients. **Conclusions:** The current results suggest that adequate endocarditis prophylaxis to prevent bacteremia may not be carried out in patients undergoing dental treatment and may occur spontaneously in patients with poor dental care. Both situations require new strategies to avoid such severe consequences.

## 1. Introduction

Despite all preventive and therapeutic efforts, infective endocarditis (IE) continues to pose a major health risk. The incidence is estimated at 13.8 patients per 100,000 people per year and mortality at around 66,300 deaths worldwide [1]. Demographic changes have led to a change in the risk pattern and now also affect high-risk patients such as electrophysiology device carriers, dialysis patients and immunosuppressed patients due to a variety of underlying diseases or their prolonged treatment [2].

There are controversial studies on the benefits of antibiotic prophylaxis to reduce the risk of endocarditis prior to dental procedures in high-risk patients [3,4,5]. Nevertheless, several studies have found that dental status and dental procedures have the potential to cause bacteremia leading to the development of IE, especially in high-risk patients [5,6,7]. The new 2023 guidelines on infective endocarditis therefore recommend appropriate antibiotic prophylaxis in patients at high risk of IE who undergo high-risk dental procedures, such as tooth extractions, oral surgery and dental procedures involving manipulation of the gingiva [1].

Dysbiosis of the oral microbiota in terms of periodontitis has been recognized as an emerging pathogenic risk factor for the development of cardiovascular diseases (CVDs). By entering the bloodstream, oral bacteria can adhere to heart valves, especially in patients with preexisting cardiac disease or after heart surgery. While increased amounts of Streptococcus were found in saliva and subgingival plaques in rheumatic heart disease (RHD), Streptococcus mutans and Aggregatibacter actinomycetemcomitans were identified as major causative agents of CVDs. In addition, Streptococcus sanguinis, Streptococcus oralis and other Streptococcus sp. were detected in heart valves of patients with aortic stenosis [8,9]. Neutrophils play a significant role in maintaining oral health and controlling periodontal disease [10]. In the absence or dysfunction of neutrophils, patients can develop severe forms of periodontitis at a young age. Excessive or hyperreactive neutrophils can lead to an imbalance in host-microbe interactions in the periodontium, resulting in dysbiosis and inflammatory tissue breakdown. Periodontal pathogens can redirect neutrophil responses in favor of their microbial community and to the detriment of the host [11].

On the other hand, there is not yet sufficient knowledge about the risk to patients with poor or desolate dental status. Although it has already been shown that commonplace activities like tooth brushing and chewing can trigger bacteremia in patients, especially if they suffer from oral diseases [12,13], the effect of such repeated daily bacteremia on the development of endocarditis has not yet been clarified. Nonetheless, there is an overall relationship between heart disease and oral inflammation [14], alongside a high prevalence of oral diseases, especially in patients with severe heart diseases like heart insufficiency, after heart transplantation or with assistive devices [15]. It has also been shown that dental behavior, as well as the perception of their oral conditions, is reduced in patients with severe heart diseases [16]. Generally low health literacy, such as smoking, alcohol consumption, lack of exercise, and poor food habits can increase the risk of both cardiac and dental disease. Some studies report a correlation between the degree of chronic oral bacterial colonization and cardiac disease. Taken together, poor oral health appears not rare in patients with heart diseases and brings a potential but unclear risk to the development of infectious endocarditis.

Against this background, the current study used the data from our Brandenburg Endocarditis Register (B.E.R.) [17], aiming at the evaluation of associations between dental treatment as well as poor oral health and IE. The focus of interest was also whether typical oral germs were identified as causative agents in patients with proven IE in whom a connection with dental treatment or a desolate dental status could be assumed. Therefore, two hypotheses were formulated as follows: (I) Previous dental treatment and poor dental status are commonly reported in patients with IE, indicating an association between those conditions. (II) Oral germs are more likely to be verified in IE patients with poor oral status and/or previous dental treatment.

## 2. Material and Methods

### 2.1. Study Design and Brandenburg Endocarditis Registry

We initiated a prospective multicenter Brandenburg endocarditis registry (B.E.R.) in March 2020. The approval required according to the European Hospital Regulation for the registry foundation has been obtained from the responsible Ministry of Health. An ethical vote was previously obtained from the corresponding Ethics Committee prior to the start of data collection (E-01-20191007, dated 20 January 2020, Ethics Committee of Brandenburg Medical School, Germany). The endocarditis registry was entered into the German Clinical Trial Registers and WHO (DRK S00023423). Written informed consent was obtained from all included patients. The only inclusion criterion was a confirmed endocarditis diagnosis according to the revised Duke criteria of the ESC 2015 Guidelines. Exclusion criteria were refusal or recall of consent to participate in the registry as well as a later disproval of the endocarditis diagnosis due to negative histopathological and intraoperative valve findings.

### 2.2. Data Collection

The data of 530 patients from cardiology and cardiac surgery departments with proven bacteria from blood cultures were analyzed for this study. Data were collected from 5 centers. The inclusion rate was 98%. The collected data included baseline, comorbidities, retrospective identification of origin of infection, previous infective endocarditis (IE), risk profile, and detailed diagnostic findings, including computed tomography, transesophageal echocardiography, microbiology, histopathology, and laboratory data. Furthermore, information about therapeutic procedures, including type, duration and dosage of antibiotic therapy; type and extent of surgery, if applied; perioperative course and complications due to IE; complications due to therapeutic procedures; time from symptom beginning to diagnosis; time from diagnosis to referral to cardiac surgery, if performed; and mortality were collected. Moreover, a potential predisposition for IE, a previous time-related exposition, as well as indication and guideline-based implementation of antibiotic prophylaxis were determined. The respective proven pathogens that were isolated by standard microbiological analysis from at least two aerobic and anaerobic blood culture pairs according to current guidelines of the respective patients were compiled and analyzed. Upon admission, patients were asked about the presence of dental implants, crowns, dental bridges or artificial dentures or known dental problems such as root abscesses. In case of conspicuous dental findings, a dentist was consulted to assess the necessity and urgency of treatment as well as to elucidate hidden infections such as root granulomas. All person-related data are pseudonymized. Follow-up duration was 12 months. In addition, a portion of the patients remained in outpatient follow-up care.

### 2.3. Dental Health-Related Parameters

Two dental health-related outcomes were considered for the current study. Firstly, previous dental therapy was evaluated. All patients who had undergone tooth extraction, dental implantations, root resection, root canal treatment or treatment of deep periodontal pockets within 3 months prior to IE diagnosis were included in the group TREAT.

Secondly, desolate dental status was considered as an outcome variable. Dental status was initially assessed by the treating cardiologist, cardiac surgeon or anesthesiologist. A desolate status was assumed if destructive dental caries of several teeth, rotten stumps, or clinical signs of progressed periodontal diseases like swelling, bleeding and/or tooth loosening were visible. If possible, a dentist was consulted for these patients. In some instance patients required acute surgery so that any further assessment by a dentist had to be postponed. These patients were included in the group DESOLATE. In most cases, the cause of the development of endocarditis could be determined in patients with a healthy dental status without previous dental treatment (CONTROL group). These included previous urogenital sepsis (12.12%), pulmonary infection (18.94%), bone and joint infections (e.g., spondylodiscitis) (9.09%), skin and soft tissue infections (16.67%), intra-abdominal infection (6.7%), infected foreign material (port, dialysis or central venous catheter, pacemaker) (30.87%), intravenous drug abuse (2.65%) and in one case, a complication of an infected piercing.

### 2.4. Statistical Analysis

In the first assessment, all patients were identified who underwent dental treatment less than three months before surgery (TREAT). Furthermore, patients with a desolate dental status at the time of surgery were identified (DESOLATE). Both groups were compared with CONTROL patients who were treated for IE but had neither dental treatment nor a desolate dental status prior to surgery.

Propensity score (PS) matching was performed. Matching parameters were age, gender, EuroSCORE II and cardiac surgery history. The statistical analysis was performed using “R” version 4.1.1 [18]; PS was performed between two respective matched pairs. TREAT patients and DESOLATE patients with the respective CONTROL patients using the matchit package [19]. Numerical variables were compared between both matched groups using Student’s t-test for normally distributed data and the Mann–Whitney U test otherwise. Categorical data were compared using Fisher’s exact test and the chi^2^-test, respectively.

## 3. Results

### 3.1. Result of Endocarditis Patients After Dental Treatment

#### Baseline and Risk Factors

A total of 13.58% (72 of 530) patients were diagnosed with endocarditis on average 48 ± 38.4 days after dental treatment. These were predominantly male (70%) with a mean age of 64 ± 13 years. Of these, 40.3% (29/72) had previously undergone cardiac surgery. 41.7% had a prosthetic valve (30/72, surgical valve replacement or transcatheter valve implantation). The proportion of pacemaker/ICD or CRTD recipients was 9.7% (7/72). 5.5% had a port or dialysis catheter. Coronary heart disease was known in 37.5% and diabetes mellitus in 23.6%. Endocarditis recurrence was present in 6.9% and early endocarditis (within 12 months of valve implantation) in 4.2%. Mean EuroSCORE II was 12.5% ± 14.7, and mean BMI was 28.1 ± 5.3 kg/m^2^. The result mainly includes aortic valve interventions. However, some concomitant procedures, mitral and tricuspid valve replacement or repair, are included.

### 3.2. Comparison of Endocarditis Patients After Dental Treatment with CONTROL Group After Propensity Score Matching

After PS matching, the data of 59 patients per group were analyzed. These did not differ significantly between the TREAT and CONTROL groups with regard to preoperative baseline (Table 1).

Early endocarditis was observed significantly less frequently in TREAT (3.57% vs. 17.54%, *p* = 0.029) (Table 2).

Furthermore, streptococcus mitis (26.92% vs. 3.51%, *p* = 0.001) and other streptococci (36.54% vs. 10.53%, *p* = 0.003) were identified significantly more frequently in TREAT. Moreover, staphylococci (1.92% vs. 14.04%, *p* = 0.033) and Enterococcus faecalis (9.62% vs. 26.32%, *p* = 0.045) were detected significantly less frequently in TREAT compared to CONTROL (Table 2).

After endocarditis diagnosis and prior to surgery, pleural effusion was detected significantly more frequently in TREAT (46.43% vs. 23.64%, *p* = 0.021) (Table 3).

**Figure 1:**
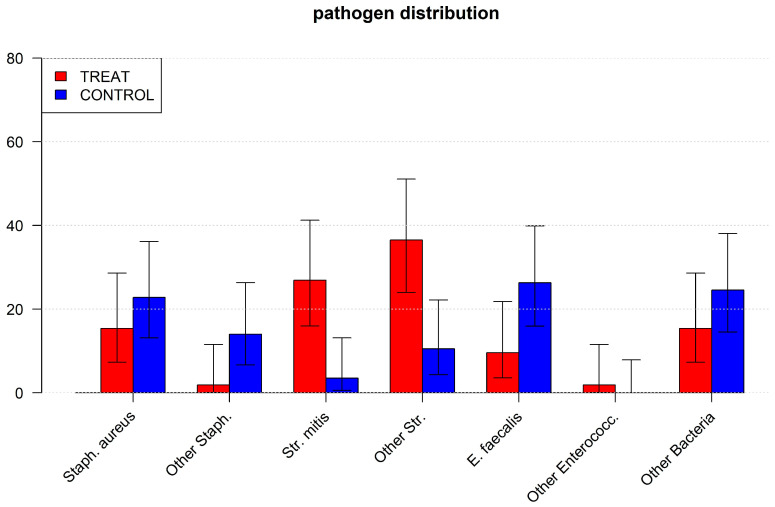
Figure 1 shows the distribution of bacteria in endocarditis patients in the CONTROL and TREAT groups after propensity score matching. Control: patients with endocarditis and normal oral and dental health without previous dental treatment; TREAT: patients with endocarditis and previous dental treatment; Staph. aureus: Staphylococcus aureus, Staph.: staphylococci; Str. mitis: Streptococcus mitis, E. faecalis: Enterococcus faecalis; Enterococc.: enterococci.

The difference in other preoperative or postoperative complications and mortality was not significant between the two groups (Figure 1) (details in Table 3 and Table 4).

### 3.3. Result of Endocarditis Patients with Desolate Dental Status

#### Baseline Situation and Risk Factors

Endocarditis was diagnosed in 10.38% (55 of 530) of patients. The patients were mostly male (78.18%) and had a mean age of 58.49 ± 12.7 years. The mean EuroSCORE II was 9.67% ± 11.28%, and the mean BMI was 27.66 ± 6.31 kg/m^2^. In 29.09% (16/55), it was a redo surgery. In addition, 30.9% had a prosthetic valve (17/55, surgical valve replacement or transcatheter valve implantation), 5.45% (3/55) had a pacemaker/ICD or CRTD and 7.27% (4/55) had a port or dialysis catheter. Endocarditis recurrence was found in 14.54% (8/55), and early endocarditis (within 12 months after valve implantation) in 9.09% (5/55). Coronary heart disease was known in 16.36% (9/55) and diabetes mellitus in 20% (11/55).

### 3.4. Comparison of Endocarditis Patients with Desolate Dental Status with CONTROL Group After Propensity Score Matching

After PS matching, the data of 48 patients each in the DESOLATE and CONTROL groups were analyzed. It was found that patients with a desolate dental status suffered significantly more from alcohol abuse (Table 5).

Again, significantly more Streptococcus mitis was detected in the blood cultures of patients with desolate dental status (Table 6).

Pleural effusion was also observed significantly more frequently preoperatively in patients with a desolate dental condition (details in Table 7).

**Figure 2:**
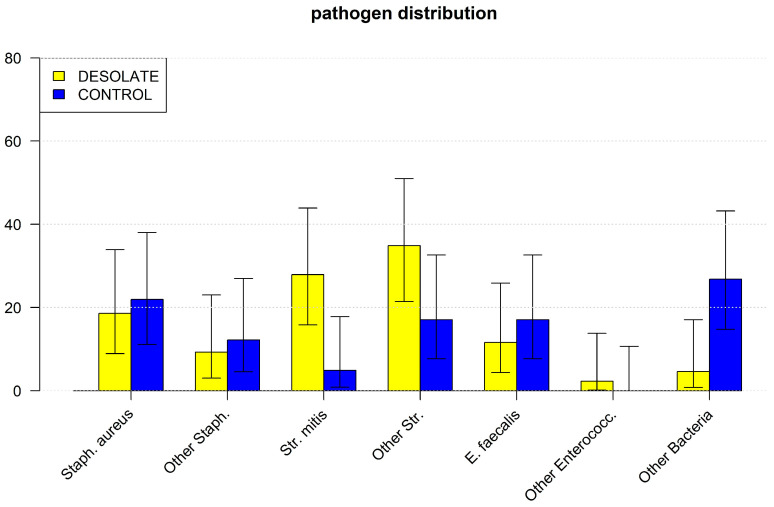
Figure 2 shows the distribution of bacteria in endocarditis patients in the CONTROL and DESOLATE groups after propensity score matching. CONTROL: patients with endocarditis and normal oral and dental health without previous dental treatment; DESOLATE: patients with endocarditis and a desolate dental status; Staph. aureus: Staphylococcus aureus; Staph.: staphylococci; Str. mitis: Streptococcus mitis; E. faecalis: Enterococcus faecalis; Enterococc.: enterococci.

Compared to the CONTROL group, patients with desolate dental status carried port and dialysis catheters significantly less often. No significant difference was observed between the groups for the other risk factors, preoperative and postoperative complications and mortality (Figure 2) (details are shown in Table 5, Table 6, Table 7 and Table 8).

## 4. Discussion

This study clearly demonstrated that Streptococcus mitis, a typical, commensal oral bacterium, is a potential main cause of endocarditis in patients with infective endocarditis. It has also been shown that bacteremia with these bacteria appears quite frequently and occurs both during dental treatment and in patients with poor dental status [20]. Thereby, Streptococcus mitis is a very common oral bacterium, as it is involved in dental biofilm formation and is therefore not solely connected with oral diseases, although it has a high pathogenic potential outside the oral cavity [21]. Fortunately, endocarditis of this origin does not appear to lead to a worse outcome than in patients with other causes. Early mortality and the vast majority of complications showed no relevant differences. In a recent study, we were able to show that Streptococcus mitis appeared to be less virulent than Staph. aureus or enterococci as a proven pathogen [22]. Interestingly, we found that both groups had more pleural effusions than the respective control patients. We can only speculate about the causes. We hypothesize that patients with endocarditis following dental treatment have less severe chronic heart failure than the control patients and may therefore be more prone to developing pleural effusion in the early postoperative course. Another, but more far-fetched, speculation is that the inflammatory response to Streptococcus mitis may differ from that to Staphylococcus or Enterococcus. However, the whole issue can be adequately managed and does not affect the outcome. In contrast to the previous guidelines, the current one finally also addresses profound dental measures as relevant for the development of IE and recommends antibiotic prophylaxis in high-risk patients. However, this may be a problematic limitation. It is clear that the definition of a high-risk patient depends heavily on current knowledge. However, this topic in particular steadily requires adjustment upon new evidence of situations and diseases that should be considered high-risk, such as device users, immunocompromised patients or patients on chronic dialysis [6,7,8,9,10,11,12]. About ten years ago, an English analysis indicated that the number of antibiotic prophylaxes has been largely reduced after the 2008 guideline, resulting in a remarkable increase in infectious endocarditis [23]. It may therefore be appropriate to include patients with an intermediate risk profile or to consider oral inflammation and type of dental intervention alongside the health- and medication-related risk of infectious complications. What exactly this means is also controversial. Even completely healthy individuals can always be in an immunocompromised state when they enter the dental practice, e.g., with an acute infectious disease such as influenza or COVID or a severe cold or bronchitis. Temporary bacteremia can then encounter a weakened immune system, and the germs have the chance to attach themselves to the valve endocardium, quickly cover themselves with biofilm, and begin their destructive work [13]. In view of the serious consequences, it may therefore be justified to carry out endocarditis prophylaxis in all patients undergoing such therapy. One could even argue that any dental treatment involving profound manipulation of the gingiva should be prophylactically protected with antibiotics prior to the procedure. Currently, professional tooth cleaning is not considered such a procedure, but it is precisely during this procedure that all gingival recesses are manipulated, and at least temporary bacteremia is inevitable. It should be noted that in the older literature, professional tooth cleaning was indeed considered a high-risk procedure for transient bacteremia [24].

We found clear evidence that a desolate dental status causes endocarditis with Streptococcus mitis. It is known that periodontitis, i.e., a high bacterial load in close contact with chronically inflamed gingiva, causes transient bacteremia even by chewing or tooth brushing. This is caused by the inflammatory increase in permeability of the junctional epithelium [25]. On the other hand, Enterococcus faecalis, which is known to be related to endodontic infections [26] and might be expected in desolate tooth status with endodontic infections, was present but not increased in patients with desolate dental status in the current study.

The actual definition of a desolate dental condition is not clear. However, a simple look in the mouth by a healthcare professional can lead to a fairly clear confirmation of a dental condition. On the other hand, radiographs often bring a merit of information, especially apically inflamed teeth, which are clinically invisible [27]. Therefore, the clinical view alone might underscore the real burden of oral diseases in at-risk patients. However, considering the expected high periodontal burden, Streptococcus mitis is not the main germ in the context of periodontitis; as a multifactorial disease, established periodontitis is related to a complex biofilm, including Gram-negative, anaerobic bacteria [28]. However, specific diagnostics for the detection of Gram-negative anaerobic bacteria such as Porphyromonas gingivalis using oral swabs were not part of this study.

The current targeted antibiotic therapy is focused on one responsible pathogen. This may not be true. It is quite possible that the pathogen detected in the blood culture predominates in the blood, while other pathogens can have a locally destructive effect under the protection of a biofilm and thus remain undetected. After the operation, it may therefore be useful to carry out a PCR of the sample in order to detect and differentiate other pathogens. Antibiotic therapy can then be used in a more targeted manner. Hypothetically, such data can even lead to the identification of typical ‘herds’ of different bacterial strains that work together. That can then be helpful in targeting not yet identified ‘herd members’ aside from the pathogen proven by blood culture samples. either prior to surgery or, more helpfully, during antibiotic-only treatment of native valve endocarditis, i.e., in those patients who cannot be sampled and thus subjected to PCR.

Additional evidence for a decent judgment of a desolate dental status in this study may be the very high proportion of alcohol abusers in this group, indicating a generally precarious lifestyle. Although all this is easy to recognize on admission, these patients usually refuse to contact medical professionals for many years. Some have never even seen a doctor or dentist in their lives. Therefore, it is very difficult to detect and improve such dental status earlier. Thus, clear concepts of allocation for at-risk patients with potential oral diseases should be fostered. Similarly, as for patients prior to endoprostheses, patient-oriented and straightforward interventions should be elaborated, as antibiotic prophylaxis might be able to reduce acute bacteremia during dental interventions, but not transient bacteremia due to oral inflammation. Besides the correct use of toothbrushes and interdental products, there are various proactive, professional and home-based approaches to prevention in dentistry without requiring the use of chemical–pharmacological substances. Probiotics and parabiotics, such as chewing gum, toothpastes and mousses, can be used to balance oral dysbiosis and minimize the bacterial load. Glycine- and erythritol-based powders can also be applied to reduce the pathogenic bacterial load, for example, in the case of periodontitis. Postbiotics of natural origin can also reduce the bacterial load in the periodontal and implant region due to their antioxidant effect. In addition, ozone therapy can reduce mucositis, peri-implantitis, periodontitis, mucosal lesions and postoperative edema through its antiseptic effect [29].

Patients with chronic dialysis are in a particularly difficult situation. It has been proven that these patients are very susceptible to the development of infective endocarditis and much more likely to oral diseases [17,30]. This is due to the general risks of these often elderly and multimorbid patients, but also to the immunosuppression caused by dialysis and, finally, to the high probability of transient bacteremia during dialysis, routinely performed two or even three times a week, without the possibility of adequate treatment with antibiotics, as this would ultimately only lead to the proliferation of multi-resistant germs. At the very least, however, every single dental treatment in these patients, including tooth cleaning, should be accompanied by appropriate antibiotic prophylaxis [31]. In an aging society, the IE risk increases with increased valve disease and the resulting need for valve replacement. Here, not only are therapeutic procedures in multimorbid elderly patients a challenge, but in some cases also the diagnosis. Diagnostic confirmation using transesophageal echocardiography as the gold standard is not always possible, particularly in patients after mechanical valve replacement or after TAVI, for example, due to the artifacts caused by metal, and in cases of doubt, alternative procedures such as PET CT must be used in this risk group [32].

## 5. Limitations

As this study is a retrospective study, a corresponding bias cannot be excluded. The propensity score analysis was performed to partially compensate for this, but the formation of matched pairs further reduces the number of patients included. The exact nature and extent of each dental procedure could not be elucidated. It also remained elusive who received an appropriate endocarditis prophylaxis prior to dental treatment. Furthermore, in the majority of cases, the diagnosis of a desolate dental status was only made visually by the doctor who examined the patient on admission and only in a few patients by a dentist. Dental screening can also be performed by medical doctors; however, screening by a dentist appears more detailed and reliable, e.g., due to diagnostic procedures like periodontal probing, vitality testing and dental radiographs. For future examinations, a clear and standardized procedure with a reasonable risk classification should be applied. For this, a similar classification into high, moderate and low risk could be used for other patient groups with a risk of oral health-associated infectious complications.

The current findings are compiled in only one federal state in Germany. Due to the limited sample size, the results appear of high clinical interest, but cannot be readily generalized. As mentioned in the discussion, the microbiological results are limited by the diagnostic procedure and selection of bacteria. Future studies might use more comprehensive microbiological testing, including sample collection from the oral cavity, to confirm the oral situation as the cause of infection.

## 6. Conclusions

Our study clearly demonstrates that bacteremia with the typical oral pathogen Streptococcus mitis is associated with and might cause infective endocarditis in patients undergoing dental treatment and in patients with desolate dental status. The risk of endocarditis is not only increased by dental treatment but also by poor oral hygiene, especially in patients at risk. The first and certainly most important step in reducing the risk of endocarditis remains prevention. Our study also suggests that adequate endocarditis prophylaxis to prevent bacteremia may not be carried out in patients undergoing dental treatment and may occur spontaneously in patients with poor dental care. Both situations require new strategies to avoid such sad consequences, e.g., a consistent indication for endocarditis prophylaxis based on current guidelines and early detection of poor dental status and structured convincing of these patients to restore their dental status. This can be a particular challenge in the latter group due to the high number of alcohol addicts and comorbidities.

## Figures and Tables

**Table 1:** Baseline in TREAT vs. CONTROL group after propensity score matching.

Baseline	TREAT (59)	CONTROL (59)	*p*	OR	CI
Gender (female)	25.42% (15)	22.03% (13)	0.829	1.21	0.52
Age (years)	64.39 ± 13.29	64.68 ± 12.11	0.902		
BMI (kg/m^2^)	28.26 ± 4.84	27.22 ± 6.59	0.337		
EuroSCORE II (%)	12.47 ± 14.85	12.41 ± 14.64	0.982		
LVEF preoperative (%)	51.47 ± 11.9	53.02 ± 7.66	0.406		
Cancer	5.08% (3)	11.86% (7)	0.322	0.40	0.10–1.62
Long-term glucocorticoid-therapy	6.78% (4)	10.17% (6)	0.743	0.64	0.17–2.41
Autoimmune disease	3.39% (2)	6.78% (4)	0.679	0.48	0.08–2.74
Drug abuse	3.39% (2)	6.78% (4)	0.679	0.48	0.08–2.74
Alcohol abuse	4.55% (2)	15.91% (7)	0.157	0.25	0.05–1.29

CI: confidence interval; OR: odds ratio; LVEF: left ventricular ejection fraction; BMI: body mass index.

**Table 2:** Cardiovascular risk and bacterial distribution in TREAT vs. CONTROL group.

Cardiovascular Risk Profile	TREAT (59)	CONTROL (59)	*p*	OR	CI
Early endocarditis	3.57% (2)	17.54% (10)	**0.029**	0.17	0.04–0.83
Endocarditis recurrence	8.47% (5)	10.17% (6)	1.000	0.82	0.24–2.84
Coronary artery disease	37.29% (22)	31.03% (18)	0.604	1.32	0.61–2.84
Previous heart surgery	40.68% (24)	42.37% (25)	1.000	0.93	0.451.94
Presence of implanted valve prosthesis	43.1% (25)	43.86% (25)	1.000	0.97	0.46–2.03
Presence of port or dialysis catheter	18.97% (11)	32.73% (18)	0.145	0.48	0.20–1.14
Perianular abscess	37.29% (22)	23.73% (14)	0.162	1.91	0.86–4.25
Staphylococcus aureus	15.38% (8)	22.81% (13)	0.460	0.62	0.23–1.63
Other staphylococci	1.92% (1)	14.04% (8)	**0.033**	0.12	0.01–1.00
Streptococcus mitis	26.92% (14)	3.51% (2)	**0.001**	10.13	2.18–47.18
Other streptococci	36.54% (19)	10.53% (6)	**0.003**	4.89	1.77–13.53
Enterococcus faecalis	9.62% (5)	26.32% (15)	**0.045**	0.30	0.10–0.89
Other enterococci	1.92% (1)	0% (0)	0.477	3.35	0.13–84.06
Other bacteria	15.38% (8)	24.56% (14)	0.340	0.56	0.21–1.47

CI: confidence interval; OR: odds ratio; Bold type emphasizes the significant values.

**Table 3:** Preoperative complications in TREAT vs. CONTROL group.

Preoperative Complications	TREAT (59)	CONTROL (59)	*p*	OR	CI
Pulmonary edema	10.17% (6)	12.07% (7)	0.974	0.82	0.26–2.62
Acute cardiac failure	49.15% (29)	31.03% (18)	0.070	2.15	1.01–4.57
Septic embolism	30.51% (18)	37.93% (22)	0.515	0.72	0.33–1.55
Acute kidney failure	28.57% (16)	23.64% (13)	0.707	1.29	0.55–3.02
Delirium	10.71% (6)	23.64% (13)	0.120	0.39	0.14–1.11
Cardiac arrhythmia	25% (14)	12.73% (7)	0.159	2.29	0.84–6.20
Pleural effusion	46.43% (26)	23.64% (13)	**0.021**	2.80	1.24–6.32

CI: confidence interval; OR: odds ratio; Bold type emphasizes the significant values.

**Table 4:** Postoperative complications in TREAT vs. CONTROL group.

Postoperative Complications	TREAT (59)	CONTROL (59)	*p*	OR	CI
Wound healing disorder	10% (5)	2.08% (1)	0.205	5.22	0.59–46.46
Delirium	20% (10)	25% (12)	0.726	0.75	0.29–1.94
AV-Block III	12% (6)	12.5% (6)	1.000	0.95	0.29–3.19
Atrial fibrillation	22% (11)	20.83% (10)	1.000	1.07	0.41–2.82
Permanent pacemaker implantation	4% (2)	10.42% (5)	0.264	0.36	0.07–1.94
Paravalvular leakage	4% (2)	8.33% (4)	0.431	0.46	0.08–2.63
Stroke	0% (0)	8.33% (4)	0.054	0.10	0.01–1.87
Acute kidney failure	36% (18)	31.25% (15)	0.777	1.24	0.53–2.87
Dialysis dependency	30% (15)	25% (12)	0.743	1.29	0.53–3.13
Multiorgan failure	14% (7)	14.58% (7)	1.000	0.95	0.31–2.96
Low cardiac output	2% (1)	10.42% (5)	0.108	0.18	0.02–1.56
Re-thoracotomy	10% (5)	8.33% (4)	1.000	1.22	0.31–4.85
CIP/CIM	4% (2)	12.5% (6)	0.155	0.29	0.06–1.52
SIRS	6% (3)	12.5% (6)	0.313	0.45	0.11–1.90
Vasoplegia	6% (3)	6.25% (3)	1.000	0.96	0.18–4.99
Septic shock	16% (8)	8.33% (4)	0.357	2.10	0.59–7.48
Hospitalization (day)	34.84 ± 20.5	36.79 ± 22.9	0.642		
Mortality	23.73% (14)	15.25% (9)	0.353	1.73	0.68–4.38

CI: confidence interval; OR: odds ratio; CIP/CIM: Critical Illness Myopathy (CIM) and/or Critical Illness Polyneuropathy (CIP); SIRS: Systemic Inflammatory Response Syndrome; AV-Block: atrioventricular block.

**Table 5:** Baseline in DESOLATE vs. CONTROL group after propensity score matching.

Baseline	DESOLATE (48)	CONTROL (48)	*p*	OR	CI
Gender (female)	20.83% (10)	18.75% (9)	1.000	1.14	0.42–3.12
Age (years)	58.33 ± 13.24	57.12 ± 12.18	0.710		
BMI (kg/m^2^)	27.65 ± 6.52	27.11 ± 5.92	0.679		
EuroSCORE II	9.67 ± 11.4	12.28 ± 17.97	0.399		
LVEF preoperative	48.75 ± 9.74	52.48 ± 9.48	0.895		
Cancer	8.33% (4)	14.58% (7)	0.523	0.53	0.15–1.95
Long-term glucocorticoid therapy	4.17% (2)	10.42% (5)	0.435	0.37	0.07–2.03
Autoimmune disease	6.25% (3)	8.33% (4)	1.000	0.73	0.16–3.47
Drug abuse	14.58% (7)	6.25% (3)	0.317	2.56	0.62–10.57
Alcohol abuse	47.83% (22)	8.82% (3)	**0.000**	9.47	2.53–35.41

OR: odds ratio; CI: confidence interval; LVEF: left ventricular ejection fraction; BMI: body mass index; AV Block: atrioventricular block; Bold type emphasizes the significant values.

**Table 6:** Cardiovascular risk and bacterial distribution in DESOLATE vs. CONTROL group.

Cardiovascular Risk Profile	DESOLATE (48)	CONTROL (48)	*p*	OR	CI
Early endocarditis	6.38% (3)	8.89% (4)	0.711	0.70	0.15–3.31
Endocarditis recurrence	12.5% (6)	6.25% (3)	0.486	2.14	0.50–9.12
Coronary artery disease	18.75% (9)	27.08% (13)	0.466	0.62	0.24–1.63
Previous heart surgery	25% (12)	20.83% (10)	0.808	1.27	0.49–3.29
Presence of implanted valve prosthesis	25% (12)	21.74% (10)	0.897	1.20	0.46–3.13
Presence of port or dialysis catheter	12.77% (6)	32.61% (15)	0.041	0.30	0.11–0.87
Perianular abscess	27.08% (13)	35.42% (17)	0.509	0.68	0.28–1.62
Staphylococcus aureus	18.6% (8)	21.95% (9)	0.912	0.81	0.28–2.36
Other staphylococci	9.3% (4)	12.2% (5)	0.735	0.74	0.18–2.97
Streptococcus mitis	27.91% (12)	4.88% (2)	0.007	7.55	1.57–36.26
Other streptococci	34.88% (15)	17.07% (7)	0.108	2.60	0.93–7.27
Enterococcus faecalis	11.63% (5)	17.07% (7)	0.688	0.64	0.19–2.20
Other enterococci	2.33% (1)	0% (0)	1.000	2.93	0.12–73.99
Other bacteria	4.65% (2)	26.83% (11)	0.006	0.13	0.03–0.64

OR: odds ratio; CI: confidence interval.

**Table 7:** Preoperative complications in DESOLATE vs. CONTROL group.

Preoperative Complications	DESOLETE (48)	CONTROL (48)	*p*	OR	CI
Pulmonary edema	10.42% (5)	14.58% (7)	0.758	0.68	0.20–2.32
Acute cardiac failure	54.17% (26)	52.08% (25)	1.000	1.09	0.49–2.42
Septic embolism	35.42% (17)	50% (24)	0.216	0.55	0.24–1.24
Acute kidney failure	25.53% (12)	22.22% (10)	0.899	1.20	0.46–3.14
Delirium	29.79% (14)	24.44% (11)	0.733	1.31	0.52–3.30
Cardiac arrhythmia	34.04% (16)	17.78% (8)	0.124	2.39	0.90–6.32
Pleural effusion	61.7% (29)	31.11% (14)	0.006	3.57	1.51–8.45

OR: odds ratio; CI: confidence interval.

**Table 8:** Postoperative complication in DESOLATE vs. CONTROL group.

Postoperative Complications	DESOLATE 48)	CONTROL (48)	*p*	OR	CI
Wound healing disorder	10.42% (5)	14.58% (7)	0.758	0.68	0.20–2.32
Delirium	54.17% (26)	52.08% (25)	1.000	1.09	0.49–2.42
AV-Block III	35.42% (17)	50% (24)	0.216	0.55	0.24–1.24
Atrial fibrillation	25.53% (12)	22.22% (10)	0.899	1.20	0.46–3.14
Permanent pacemaker implantation	29.79% (14)	24.44% (11)	0.733	1.31	0.52–3.30
Paravalvular leakage	34.04% (16)	17.78% (8)	0.124	2.39	0.90–6.32
Stroke	61.7% (29)	31.11% (14)	0.006	3.57	1.51–8.45
Acute kidney failure	18.42% (7)	30.95% (13)	0.301	0.50	0.18–1.44
Dialysis dependency	7.89% (3)	2.38% (1)	0.341	3.51	0.35–35.32
Multiorgan failure	25.64% (10)	23.81% (10)	1.000	1.10	0.40–3.03
Low cardiac output	7.89% (3)	11.9% (5)	0.715	0.63	0.14–2.85
Re-thoracotomy	13.16% (5)	16.67% (7)	0.900	0.76	0.22–2.62
CIP/CIM	2.63% (1)	9.52% (4)	0.362	0.26	0.03–2.41
SIRS	2.63% (1)	7.14% (3)	0.617	0.35	0.03–3.53
Vasoplegia	0% (0)	4.76% (2)	0.495	0.21	0.01–4.52
Septic shock	18.42% (7)	30.95% (13)	0.301	0.50	0.18–1.44
Hospitalization (day)	29.84 ± 13.8	31.8 ± 14.8	0.522		
Mortality	13.16% (5)	23.81% (10)	0.351	0.48	0.15–1.58

OR: odds ratio; CI: confidence interval; CIP/CIM: Critical Illness Myopathy (CIM) and/or Critical Illness Polyneuropathy (CIP); SIRS: Systemic Inflammatory Response Syndrome; AV Block: atrioventricular block.

## Data Availability

Data is not available due to data protection and ethical restrictions.
